# A DNA pooling-based case-control study of myopia candidate genes *COL11A1*, *COL18A1*, *FBN1*, and *PLOD1* in a Chinese population

**Published:** 2011-03-26

**Authors:** Shea Ping Yip, Kim Hung Leung, Wai Yan Fung, Po Wah Ng, Pak Chung Sham, Maurice K.H. Yap

**Affiliations:** 1 Department of Health Technology and Informatics, The Hong Kong Polytechnic University, Hong Kong SAR, China; 2 Centre for Myopia Research, School of Optometry, The Hong Kong Polytechnic University, Hong Kong SAR, China; 3 Department of Psychiatry, The University of Hong Kong, Hong Kong SAR, China

## Abstract

**Purpose:**

We examined the relationship between high myopia and common polymorphisms in four candidate genes: collagen, type XI, alpha 1 (*COL11A1*); collagen, type XVIII, alpha 1 (*COL18A1*); fibrillin 1 (*FBN1*); and procollagen-lysine 1,2-oxoglutarate 5-dioxygenase 1 (*PLOD1*). These genes were selected because rare pathogenic mutations in these genes cause disease syndromes that have myopia, usually high myopia, as one of the common presenting features.

**Methods:**

This study recruited 600 unrelated Han Chinese subjects including 300 cases with high myopia (spherical equivalent or SE≤-8.00 diopters) and 300 controls (SE within ±1.00 diopter). A total of 66 tag single nucleotide polymorphisms (SNPs) were selected for study from these four candidate genes. The study adopted a DNA pooling strategy with an initial screen of DNA pools to identify putatively positive SNPs and then confirmed the “positive” SNPs by genotyping individual samples forming the original DNA pools. DNA pools were each constructed by mixing equal amounts of DNA from 50 individuals with the same phenotype status. Six case pools were prepared from 300 cases and six control pools from 300 controls. Allele frequencies of DNA pools were estimated by analyzing the primer-extended products with denaturing high performance liquid chromatography and compared between case pools and control pools with nested ANOVA.

**Results:**

In the first stage, 60 SNPs from the 4 candidate genes were successfully screened using the DNA pooling approach. Of these, 6 SNPs showed a statistical significant difference in estimated allele frequencies between case pools and controls at p<0.10. In the second stage, these “positive” SNPs were followed up by individual genotyping, but failed to be confirmed via standard single-marker and haplotype analyses.

**Conclusions:**

Common polymorphisms in these four candidate genes (*COL11A1*, *COL18A1*, *FBN1* and *PLOD1*) were unlikely to play important roles in the genetic susceptibility to high myopia.

## Introduction

Myopia is the commonest ocular disorder in the world. In general, it is more prevalent in Oriental populations (60%–80%) than in Caucasian populations (10%–25%) [1]. Subjects with high myopia, usually defined as −6.0 diopters (D) or worse, are more vulnerable to ocular pathologies later in their life, such as cataract, glaucoma and retinal detachment [2]. Myopia is a complex disease with contribution from genetic factors, environmental factors and their interactions [3,4]. Genetic association studies are usually used to identify myopia susceptibility genes, which tend to have small effect size [4,5]. Genome-wide association studies generate hypotheses for subsequent follow-up and are the method of choice, but are still beyond the reach of many research groups in terms of the cost. Another popular approach is to examine candidate genes, which are usually selected on the basis of their biology and function [4,6]. Genes underlying heritable disease syndromes with myopia as one of the common presenting features can be selected as myopia candidate genes for study [4,6].

Stickler syndrome is an autosomal dominant disease affecting types II and XI collagen expressed in vitreous and cartilage, and has highly variable clinical features affecting the eye, the ear and joints [7,8]. In particular, Stickler syndrome types 1 and 2 (STL1 and STL2) have myopia and abnormal vitreous while type 3 is a non-ocular form of the syndrome. STL1 is caused by mutations in the collagen, type II, alpha 1 (*COL2A1*) gene while STL2 arises from mutations in the collagen, type XI, alpha 1 (*COL11A1*) gene. Interestingly, *COL11A1* mutations are also known to cause Marshall syndrome or Marshall/Stickler syndrome, which both have myopia as a common feature [7,8]. Knobloch syndrome is an autosomal recessive disease characterized by high myopia, vitreoretinal degeneration and occipital encephalocele, and is caused by mutations in the collagen, type XVIII, alpha 1 (*COL18A1*) locus [8-10]. Marfan syndrome is an autosomal dominant disorder of connective tissue with major manifestations affecting the ocular, skeletal and cardiovascular systems [11,12]. The major ocular abnormalities are lens dislocation and high myopia due to increased axial length. Marfan syndrome is classically caused by mutations in the fibrillin 1 (*FBN1*) gene. Ehlers-Danlos syndrome is a heterogeneous group of genetic disorders with major clinical features of skin hyper-extensibility, atrophic scarring, join hyper-mobility and generalized tissue fragility [11]. Type VI or the kyphoscoliotic form of Ehlers-Danlos syndrome is autosomal recessive in nature with added clinical features of kyphoscoliosis (a form of curved spine) and scleral fragility [11,12]. High myopia is also a common feature [13]. The kyphoscoliotic form of Ehlers-Danlos syndrome is caused by mutations in the *PLOD1* gene, which encodes the enzyme procollagen-lysine 1,2-oxoglutarate 5 dioxygenase-1 (PLOD1; also known as lysyl hydroxylase 1) responsible for forming cross-links in collagens via hydroxylysine-based pyridinoline.

The genes responsible for these syndromes are expressed in various parts of the eye. These disease syndromes are caused by rare pathogenic loss-of-function mutations that are not found in healthy individuals. The mechanisms leading to the common occurrence of myopia, usually high myopia, in these syndromes are not well established. We hypothesized that common polymorphisms in these genes could be predisposing genetic factors for high myopia [4,6]. Indeed, common polymorphisms in *COL2A1* – the causative gene for STL1 – have been found to be associated with myopia in two family-based association studies [14,15]. In this study, we evaluated *COL11A1*, *COL18A1*, *FBN1*, and *PLOD1* (Table 1) as candidate genes for high myopia in a Chinese population with a case-control study approach.

**Table 1:** Summary of tag SNPs in the *COL11A1*, *COL18A1*, *FBN1*, and *PLOD1* genes.

**Gene**	**GeneID**	**No. of exons**	**Chromosmal location**	**Region captured**	**No. of tSNPs selected**	**No. of SNPs captured at mean r^2^=?**
*COL11A1*	1301	68	1p21	238 kb	8	85 (r^2^=0.969)
*COL18A1*	80781	43	21q22.3	115 kb	30	81 (r^2^=0.956)
*FBN1*	2200	66	15q21.1	243 kb	19	142 (r^2^=0.925)
*PLOD1*	5351	19	1p36.22	47 kb	9	16 (r^2^=0.980)

We performed the study with an initial screen of DNA pools to identify putatively positive single nucleotide polymorphisms (SNPs) and then confirmed the “positive” SNPs by genotyping individual samples forming the original DNA pools. The initial screen of DNA pools was to cut down the time and cost involved in sample-by-sample genotyping [4,16]. DNA pools were constructed by mixing equal amounts of DNA from many subjects with the same disease status. In the present study, “case pools” were prepared from individuals with high myopia (cases) and “control pools” from emmetropes (controls). Allele frequencies of DNA pools were estimated by analyzing the primer-extended products with denaturing high performance liquid chromatography (DHPLC) [17] and compared between case pools and control pools with a proper statistical method, nested ANOVA (ANOVA) [18].

## Methods

### Subjects

This study recruited 600 unrelated Han Chinese individuals including 300 cases with high myopia (spherical equivalent or SE ≤-8.00 D for both eyes) and 300 control subjects (SE within ±1.00 D for both eyes). The study was approved by the Human Subjects Ethics Subcommittee of the Hong Kong Polytechnic University and adhered to the tenets of the Declaration of Helsinki. Written informed consents were obtained from all participating subjects. Eye examination for all participants was conducted in the Optometry Clinic of the University, blood samples were collected and DNA was extracted as has been described previously [19]. Of particular relevance to this study was the exclusion of subjects who showed obvious signs of ocular disease or other inherited disease associated with myopia (e.g., Stickler syndrome, Marshall syndrome, Knobloch syndrome, Marfan syndrome, Ehlers Danlos syndrome, etc).

### Construction of DNA pools

A PicoGreen method (Invitrogen, Carlsbad, CA) was used to quantify accurately all DNA samples in accordance with the manufacturer’s protocols. The DNA samples were then diluted to 5.0±0.3 ng/μl, and then mixed in equal volumes to construct DNA pools. DNA from 50 distinct individuals sharing the same phenotype was mixed to construct a single pool. In total, six case pools were constructed from 300 cases, and six control pools from 300 controls.

### Tag SNP selection

Four candidate genes were investigated in this study: *COL11A1*, *COL18A1*, *FBN1*, and *PLOD1* (Table 1). With the Tagger program [20], the following criteria were used to select tag SNPs from each of the gene of interest and its adjoining genomic region (3 kb upstream and 3 kb downstream): pairwise tagging algorithm, r^2^≥0.8 and minor allele frequency (MAF) ≥0.10. The Han Chinese genotype data from the International HapMap Project database (release 23a, phase II) were used for tag SNP selection. In total, 66 tag SNPs were selected from these four candidate genes and screened by the DNA pooling strategy (Table 1).

### Estimation of allele frequencies in DNA pools

Genomic DNA (individual or pooled) was amplified for each SNP with a touchdown protocol in a 15-μl reaction mixture, which contained 0.1 or 0.3 μM of each primer, 1.5 or 2.5 mM MgCl_2_ (Table 2), 0.2 mM of each dNTP, 25 ng of DNA and 0.2 unit of DNA polymerase (HotStarTaq Plus; Qiagen, Hilden, Germany) in 1× PCR buffer supplied by the manufacturer. All primers were designed using the OLIGO software (version 6.57; Molecular Biology Insights, Cascade, CO). A thermal cycler (GeneAmp PCR System 9700; Applied Biosystems, Foster City, CA) was used for the touchdown PCR: (1) initial denaturation of 5 min/95 °C; (2) 6 touchdown cycles of 30 s/95 °C, 45 s/62 °C or 64 °C (initial annealing temperature, Table 2) with a decrease of 1 °C per cycle, and 45 s/72 °C; (3) additional 38 cycles of 30 s/95 °C, 45 s/56 °C or 58 °C (final target annealing temperature, Table 2), and 45 s/72 °C; and (4) final extension of 7 min/72 °C. PCR products were then purified using exonuclease I (New England Biolabs, Beverly, MA) and shrimp alkaline phosphatase (GE Healthcare, Piscataway, NJ).

**Table 2:** *COL11A1*, *COL18A1*, *FBN1* and *PLOD1* SNPs: PCR primers and conditions, primer extension (PE) primers, ddNTP added, and buffer B concentration ranges for DHPLC

** **	** **	** **	** **	** **	** **	** **	**ddNTP**				
SNP*	**Forward primer (5′>3′)**	**Reverse primer (5′>3′)**	**Primer (µM)**	**Mg^2+^ (mM)**	**Tm (°C)**	**PE primer (5′>3′)**	**G**	**C**	**A**	**T**	**DHPLC buffer B**
*COL11A1*											
rs2061705	GAAACCCTGCCTCTACTAAAAG	GGGCAAATCTTAGGAACTTACAAAA	0.1	1.5	64 to 58	CAACGTTTTCTGTGAAATTTTATAAAGAC		√		√	27–33
rs2615987	TAAGAGAAAAAAAACCAACAGCCTAT	AACACCAGAATTTGAACAGTAAGG	0.1	1.5	64 to 58	TACAGAATTAACCATAATTGAACAGC	√		√	‡	27–33
rs17127311	CATAAGGAATAAAGGCATAAGAACATC	AGAGATTTAGAAGGGTCAAGATG	0.1	1.5	64 to 58	AGGAAAAAGCAAAGCAAAGTGATG	√		√		23–29
rs12143740	ATTAGGTGAGACAGATGAAAAATTATTG	TATTACCCACTTTATGTTACTGTCC	0.1	1.5	64 to 58	GATAACTGAAAGAGGATGGGGA	√		√		22–28
rs4908273	AAGCAGGTGTAGGCGTAAGT	TAATGAGTTGGGAAGGGAAAGTA	0.3	2.5	62 to 56	TATTATTGGTTTGGGAATTTCTTTTACA	√		√		31–37
rs11164630	CTCTTTGGGTGTATCTGTGTTTA	TATATGCCCTAACTCCACAACTA	0.1	1.5	64 to 58	CAAATATAATACACAGTTGATTAGATAGC	√		√		28–34
rs9659030	CTCCTTGTTTTCAGTGTGCTTC	CCTTAAAGTAAATACCTTAGTGGAAAAAA	0.1	1.5	64 to 58	CATATGGTGGACTGTTATTAAGAGT	√		√		27–33
rs1241209 †	GTCCCCATCCAAATCTCATC	GCAAGGAGGAACAAGTCACAT	-	-	-	ATGATAGGGGCAAGTCATTCAAA	-	-	-	-	-
*COL18A1*											
rs11089001	ATTCCCTCGTATGGTGCTGT	GCCTTAGACCCCAAACTCC	0.1	1.5	64 to 58	TTGGATAAGAGGAGCACCC	√		√		21–27
rs2026885	ACAGGGTCAAGTTTCAGCAG	ATGGAAGGAAGGTCTCTCTC	0.1	1.5	64 to 58	ATGCCAGACGGCGTGAAG		√	√		19–25
rs1004133	GTTTTGTGAGTCCCCATTGC	AAAGGCTGACCATAAACAATAAAAGTA	0.1	1.5	64 to 58	GTCGTGTTTCTCTGGGAAC	√		√		22–28
rs2838907	GCCCTTTCTGTATTGGATGC	CACAACATTCTCAGTCATTTTCACA	0.1	1.5	64 to 58	CAAAGGAATGGGCTCCAGT	√	√			20–26
rs2838913	TCCGTTGTTGTAGCCACTGT	CCTTTTCTCTCTTCCCTACG	0.3	2.5	62 to 56	CACCTCCTTCACCACTTCA		√		√	26–32
rs2838916	ATTTATCAGGCGTTTTGGGTGTA	AATGCTGAATAATGCTCCTGTTG	0.3	2.5	62 to 56	GCCACTTGGTTGCATTGTGAAAT	√			√	26–32
rs2838917	GTCCTGGTATTTATCTCAACTTCAT	GCTTACTGACTCCACATTACG	0.3	2.5	62 to 56	AAGCTGCTTTTTATTATTTGTGACC	√		√		29–35
rs2838920	TCAAAGGTTCTCTCCACTCG	AGTGTTGGGGGCTGTGTTG	0.1	1.5	64 to 58	CCTTGCACAGCCAGAAACG		√		√	22–28
rs8131523 †	AGGACGGCGACTCACAGAT	AGGACAGAATAGATGGCAGAAG	0.3	2.5	62 to 56	CTGGCCTGACGCACCTCG	-	-	-	-	-
rs2838922	TCCCCACCTTTTCACAGTTG	AAACCCCATCTCTACTAAAAACACAA	0.1	1.5	64 to 58	GACAGCATTTAGTTTGAGTGACTTT		√		√	28–34
rs2838923	CTCTGTCTTTTCTCCTCTGTTGA	CAGAAAGCAAACGCACAGAC	0.1	1.5	64 to 58	AGACTCCTGAGGTGCAGAC	√		√		23–29
rs11911327	GTGTGCATCTTGAGTGCTGT	GGTCCTGGCTGCTACTGAA	0.1	1.5	64 to 58	GGCCTCGGGCCTCAAAGA		√		√	21–27
rs8126757	GTTTGAGGAGCGTGTGGTG	TCCAATCGTTTCCAGTCCTTC	0.1	1.5	64 to 58	CTGCTACTGAAGTCTCCTGA		√		√	24–30
rs2838927 †	TTGAGGAGCGTGTGGTGAG	GAGTTGGGCAGTGGTGATG	-	-	-	GACCGAGGCCACCGGTTTC	-	-	-	-	-
rs11702782	ATCACCACTGCCCAACTCTA	AGAGGAGCCCGAAACAAAAG	0.1	1.5	64 to 58	CACTTTCCACCTCTGGATC	√		√		24–30
rs2026887	CCAAGATGAAAGGCCAGAGT	AAGAGGAGACAGGAAGTGGA	0.1	1.5	64 to 58	ACGGGGAGCTGTGGGTGA		√		√	21–27
rs8129539	CTAGAATAAGTATGAGAAACCCCAAAT	CTCACAAGGTCAGAAGGAATCAA	0.1	1.5	64 to 58	AGTGTACACAGATGTTAATCCTCTT	√	√			28–34
rs8133622	GACAGAATACTCCCTAAATCAACTA	GAACCAAAAGACCTCAATGTGC	0.1	1.5	64 to 58	TAAATTGAATTTCAAGGAACCCAGA	√		√		28–34
rs7279077	CACACAGGTTTCTGCATGGA	GCTCTGCAAAAAGAAAGACAGCT	0.1	1.5	64 to 58	CGTTTGGGAATGAGTGAACC			√	√	22–28
rs2236454	TGGGTGAGTGAGTGAGATTG	TGGCTTTGCTTTCTTTGAACTTC	0.3	2.5	62 to 56	GAGATCCAGGAAACTCCCC		√		√	22–28
rs2236457	TGGGGTGAGTGACATCTGG	GAGGGTGGTTTCTGGTGTTTAT	0.1	1.5	64 to 58	AATGGTATCGCAGCTTCCCAGT	√		√		26–32
rs2230687	CAAACCAAGCAAGTCTCCAC	CACCACATCCAGAAAAGAGC	0.1	1.5	64 to 58	GCTCCCCGCGCCACCCCC	√	√			20–26
rs9977482 †	ATGTGCATGTGTTTGAGTGTGTG	TGTGTATGCAAGTGTATCCACGT	0.1	1.5	64 to 58	TGTGCATGTGTGGTATGTGTAC	-	-	-	-	-
rs3818661 †	AGGGAGTGTGGGGTTAGGT	GCAGGATGAGATGGCAGAG	0.1	1.5	64 to 58	CAACCCTACCGATGGGCGCTC	-	-	-	-	-
rs2236475	ACTGTCCTCCCCCAAGAAC	TGATGATTACCAGACACCTTCC	0.1	1.5	64 to 58	CCCACTGCCCTGTCTGCC		√		√	21–27
rs7279445	CTGTAAGTATGACGAGGGTAGA	CAAACCCACACCAGCCTCT	0.3	1.5	62 to 56	GGGAGCGTCTCTTGTAAGC	√		√		23–29
rs3753019	AGTAAGTCCCAGCCTGTGCA	GCAGAGACCTGACAACAGTG	0.1	1.5	64 to 58	CCATCAGTCCTGAAAGACTC	?	√	?	√	22–28
rs2838951	AAGTGGGCTTGGCTCCATC	CATACGCTGCCAGGTCAGA	0.1	1.5	64 to 58	GTTCCTCAAGGATGTGACAG	√	√			
rs7499	GCTGCCATCACGCCTACAT	CTCTTTGGCTTCCTTTTATTTCTTG	0.1	1.5	64 to 58	TAGCCACCGCCTGGATGC	√		√		22–28
rs1051296	ATGGTCCTGTCTGTCCTTCT	GCACATACCAAGGCCAGCA	0.3	1.5	62 to 56	TCCCCTCCGGGCTGGCAC		√	√		21–27
*FBN1*											
rs6493334	GCTCATAGTTCCCACAGTTTTTC	CTGGCTGCTGTAACTGGAG	0.1	1.5	64 to 58	TCAGCATGTCCATCATCACCAT			√	√	24–30
rs6493333	TAACTTGCCCAGGTCTCTCTA	GGCTGCTGTAACTGGAGAC	0.3	1.5	62 to 56	AGGTACCCTGGGAATGTATC		√		√	22–28
rs6493331	GCCACCTTTCAGAGACCATTTT	ACCCCATCTCTACTAAAAATACAAAAAA	0.1	1.5	64 to 58	TGAACTTTACTTACTTTTTATGTATCTTTAAT	√			√	31–37
rs2247876	TGGAAAGGGGGTCTGGTATT	CTTGTTGGACTAAATAATACTGTGTGA	0.1	1.5	64 to 58	ACCTCGGCTCTCACATCTG		√		√	22–28
rs16961274	CTGAGGAGATGTAGGCAAATG	CAGGCTCAAATAGGAAATAGTGT	0.1	1.5	64 to 58	GAGTTGAGGCCACTGACCT	√	√			20–26
rs12438332	ATCTGAGAATGAGCACACCAATA	CTAGCCCTCCCATCAAAACA	0.1	1.5	64 to 58	TGCATGCTTTTACCTAACTTCCT	√	√			25–31
rs1807301	GGGTGTCCTTTGTTGTGTGA	GTCAGGAGGCTCTTATTTCATATA	0.1	1.5	64 to 58	AGTTAATGGAGCAGGGTCAG		√		√	22–28
rs16961220 †	AGAGCAGAGAGTGGTTTGTG	TCTGTGCAAGTTGTAAAGAGAAG	0.1	1.5	64 to 58	CAATGTAAAGGTCTATTATGCATTACA	-	-	-	-	-
rs16961207	GTTTTCTCTCATTTCTCCATCTG	GCATAACAGCCAACAGACTTTC	0.1	1.5	64 to 58	CTTGGGTAGGAATTTAACACAGAA		√	√		25–31
rs12915677	CTTTGCCATTCCTCTCTCCA	CTCCAGACCAGAAACAGTAAAAAA	0.1	1.5	64 to 58	TCAGTACTGGGGATTGTCTAAAA		√		√	25–31
rs683282	GAAGTTGGGGATAGAAGATAAGA	CTGTGGAGACTATTTGGCATTG	0.1	1.5	64 to 58	GTCAATGTACAGCAATACACCAT	√		√		28–34
rs16961118	TATTTATTCACTACCTCCTCCACAA	GATCACACCACTGCTCTCAA	0.1	1.5	64 to 58	GTGCTATTCACTGATACAGGGTT	√		√		25–31
rs6493328	CTGTCATTTTTTTCTCTTCTTCTGC	GACATCCTCTAATAAACATCCTGAA	0.1	1.5	64 to 58	GTGTGGGTCAGGCTATTGAAAA		√		√	25–31
rs17361868	CCGTGTGTGTGTTGGTAAGAAA	GCCTGGAGAAAACTGAAGAC	0.1	1.5	64 to 58	CAATAAAGCCCAATGGTAACAAAC	√		√		23–29
rs11635140	GCAGACACGCTCCAACAATA	CTGTTAGGTAAAATACTGC	0.1	1.5	64 to 58	TATTCTGAGAGATCCCACAGTG		√		√	26–32
rs11070643	CTATACAAGATATGAAAATAATGTGCATC	AATGTAAGTTTCCAGTCACTGCA	0.3	2.5	62 to 56	GACTTACTAAAAATACTAGCAAAAGAAAATAT	√		√		28–34
rs3825963	GAATAGGAAAATGAAGGTTGAGTG	AGAAACACAGGGGGCAGATT	0.1	1.5	62 to 56	GGTGATTTAGGGAGCTTCCA		√		√	22–28
rs10519174	TCTATATGATGAAAGAAAAAGCCTGTA	GAATTTTACAAGAACACTAAGACTCAT	0.1	1.5	62 to 56	AAAGATTAACCCACAAATCAGGAG		√		√	24–30
rs1467953	CTAGAGACAGGTTTTGCCATGT	CTTTAGTCGTGATTCTTCTTAGGAT	0.1	1.5	64 to 58	CAGTTTTATTAAAAGTGCCTTTAGCTATT		√	√		28–34
*PLOD1*											
rs1208984	GGAAGGAAAGGGTGACAGC	ACGGGGATAATAGAAGGAAACAC	0.3	1.5	62 to 56	AAGGCTCAAATAATAATAGTTTTCATAATAATA	√		√		30–36
rs7529452	TAAGGGGTGTTTCTCTCCAG	CACCTCCTCTTTATCTTTTCACC	0.1	1.5	64 to 58	AGGAGGATCTGGTCATTCTCTT		√		√	24–30
rs7551175	TAAGGGGTGTTTCTCTCCAG	ACCTCCTCTTTATCTTTTCACCA	0.1	1.5	64 to 58	TGACCCACCTACCTGTCTG		√		√	22–28
rs11585018	TGCTCTTGTGGTTGCTTGTC	GGTGGTGAAGAAACGGATTG	0.1	1.5	64 to 58	GCCAGGTTTCCCAGATAACT	√		√		23–29
rs2273286	GCTCTTGTGGTTGCTTGTCA	TGAGTTCCTAATGACCCCAAG	0.1	1.5	64 to 58	CAATCCGTTTCTTCACCACCTAT	√		√		25–31
rs1130529	ATCCCTGGTTAGTGCTGTCT	AGAAGCCAGATGAAGGTGTC	0.1	1.5	64 to 58	TCATCAGCGGGGCAATGAC	√		√		21–27
rs2273291	GACCAACATTTCAGGACCAG	ATATCAGAGACCAAGTGCAGTC	0.1	1.5	64 to 58	GAAGACTTCCCTGATCACATTCT		√		√	24–30
rs3818157	ACTCTTGACATAGGGGTGCT	CCTGCTGTCTGATGAACTTG	0.1	1.5	64 to 58	CTCCATCTCAGGTTTCTCAATC		√		√	24–30
rs3753579	GAGATTGTGGAGGAGGAAAC	ACACTGCCCTCTGAGTAGC	0.1	1.5	64 to 58	TGTCTGGGATGTAGCGTGC		√		√	20–26

*SNPs are arranged down the column in the order of 5′>3′ along the sense strand of the respective gene. †These six SNPs failed to be analyzed by the DNA pooling approach because of failure in PCR (rs1241209 in *COL11A1*, and rs2838927 in *COL18A1*) or PE reaction (rs8131523, rs9977482 and rs3818661 in *COL18A1*, and rs16961220 in *FBN1*) even after repeated optimization. This is the reason why specific conditions for PCR and/or PE are left blank (indicated as “-”). ‡In addition to ddGTP and ddATP, dTTP was also used in the primer extension reaction for rs2615987 (*COL11A1*) for an optimal discrimination of the extended products.

Primer extension (PE) reaction was performed in a 25-μl reaction mixture, which contained 10 μl of purified PCR product, 1.5 μM of a specific PE primer (Table 2), 50 μM of each appropriate ddNTP (Table 2) and 1 unit of Therminator (New England Biolabs, Beverly, MA) in a 1× reaction buffer provided by the manufacturer. Amplification was conducted as follows: initial denaturation of 1 min/96 °C, followed by 55 cycles of 10 s/96 °C, 15 s/43 °C and 1 min/60 °C. The WAVE Nucleic Acid Fragment Analysis System (Transgenomic, Omaha, NE) was used for DHPLC analysis of primer extended products. PE products were analyzed as described previously [21] with the following modifications: a 6% linear gradient change of the working elution buffer over a 3-min period and a different starting concentration of buffer B, which varied with the SNP being studied (Table 2).

Estimation of the relative allele frequencies in DNA pools was based on the peak heights of the PE products as analyzed by DHPLC. Each DNA pool was analyzed in three replicates, and each replicate consisted of a single PCR followed by a single PE reaction and a single DHPLC analysis. Therefore, each SNP had 36 sets of readings for 6 case pools and 6 control pools. For each SNP, a heterozygous sample was first identified by screening 10 to 40 subjects, and then analyzed in three independent runs to obtain a mean value for the so-called “k correction factor” that was used to correct for differential incorporation of ddNTPs in PE reactions as described previously [17].

### Individual genotyping

The positive findings (6 SNPs) in the initial screen of DNA pools were confirmed by genotyping the individual samples that formed the original DNA pools. The MassARRAY iPLEX Gold assay was used to genotype the samples in accordance to the manufacturer’s protocols for 5 SNPs (Table 3). Because of the multiplexing format of the MassARRAY system, these SNPs were grouped and genotyped together with SNPs of other studies by a local service provider. One SNP (rs2838922) could not be grouped together with other SNPs for the MassARRAY system, and was genotyped by the method of restriction fragment length polymorphism (Table 3). The fragment was amplified using touchdown PCR as described above with the following specific conditions: 0.1 μM of each primer, 1.5 mM MgCl_2_, 64 °C as the initial annealing temperature and 58 °C as the final target annealing temperature. Overnight digestion of the PCR products by TaqI (Fermentas, Vilnius, Lithuania) at 65 °C was performed according to the manufacturer’s instructions. Digested products were separated by electrophoresis in polyacrylamide gels.

**Table 3:** Primers for genotyping individual samples.

**Gene, SNP**	Primer sequences (5′>3′)*
MassArray iPLEX Gold assay (Sequenom)	
*COL11A1*, rs17127311	F: ACGTTGGATGCCTTTAGACTTCACATTCTC
	R: ACGTTGGATGGTATTAAGGAAAAAGCAAAGC
	PE primer: cccacGGAAAAAGCAAAGCAAAGTGATG
*COL18A1*, rs11911327	F: ACGTTGGATGTTTGCGTGGCTGCCTGGCCT
	R: ACGTTGGATGGACTCACAGATGCCTTTTGC
	PE primer: caTTCCCACAGCGCTGC
*COL18A1*, rs2236454	F: ACGTTGGATGCAGAAGCCAAGGACAGAAAC
	R: ACGTTGGATGATTGGGTCCGGACGGAATG
	PE primer: aaagcGATCCAGGAAACTCCCC
*COL18A1*, rs2236457	F: ACGTTGGATGGCTACAGGAGAGCACAGAAA
	R: ACGTTGGATGTCTATGACAGGAAAAGTCCC
	PE primer: aacaCCAAAATATACCACTTGGGG
*COL18A1*, rs2236475	F: ACGTTGGATGGCCAGTACCCAGGAGGAAG
	R: ACGTTGGATGTGACTGAGCCTAGCACACAC
	PE primer: ggacGGCCCACTGCCCTGTCTGCC
Restriction fragment length polymorphism	
*COL18A1*, rs2838922	F: CTGCTTCCCCACCTTTTCAC
	R: (T)_20_ CTGAGATGTGAGAATCGCTCGA

*F=forward primer; R=reverse primer; and PE=primer extension. Note that bases in lower case are added to the PE primers to allow better size discrimination of the primer extended products in the multiplex assays of the MassARRAY system.

### Statistical analysis

Ocular data were analyzed with the STATA package (version 8.2; StataCorp, College Station, TX). Subjects were classified as cases (affected with high myopia) or controls (unaffected). For a given SNP, the relative allele frequencies were estimated from the peak heights of the two extension products and adjusted using the k correction factor according to the method reported by Hoogendoorn et al. [17]. With the STATA package, nested ANOVA [18] was used to compare the relative allele frequencies of the case pools and the control pools. A p value ≤0.10 for the comparison between case pools and control pools was used as the threshold for following up SNPs with individual genotyping. Genotype data of individual samples were tested for Hardy–Weinberg equilibrium (HWE), and compared between cases and controls for association. The PLINK package (version 1.07) [22] was used for analysis. The linkage disequilibrium measures were calculated and plotted using Haploview (version 4.2) [23]. Haplotype blocks were constructed using the algorithm known as the solid spine of LD, which is unique to Haploview. Potential interactions among SNPs were examined using the method of multifactor dimensionality reduction (MDR) [24].

## Results

### Analysis of the ocular data

The characteristics of the participating subjects have been reported in one of our previous studies [19]. They are briefly summarized as follows. The average SE was −10.53 (range: −24.00 to −8.00) D for cases, and 0.03 (range: −1.00 to 0.88) D for controls. The average axial length was 27.76 (range: 24.62 – 31.29) mm for cases, and 23.85 (range: 21.24 to 27.71) mm for controls. These ocular data are for the right eyes. The average age was 27.7 (range: 15 to 48) years for cases, and 24.9 (range: 17 to 46) years for controls. There were more male subjects in the control group than in the case group (43.7% vs 28.3%, p=4.30×10^−5^).

### Analysis of results for DNA pools

The results are summarized in Table 4. Of the 66 tag SNPs selected for study, 6 did not give any results because of failure in PCR or PE even after repeated optimization as noted in a footnote of Table 2. For the 60 SNPs successfully analyzed, the k correction factor ranged from 0.29 to 1.56 with a mean of 1.02; it ranged from 0.83 to 1.21 for 54 SNPs (90% of the SNPs analyzed). The estimated frequencies of the first eluted alleles ranged from 0.1041 to 0.9246 for case pools, and from 0.0929 to 0.9516 for control pools. The difference (case pools – control pools) in estimated allele frequencies varied from −0.0510 to 0.0337. At a lenient threshold of p≤0.10, six SNPs gave significant results, which were followed up with individual genotyping for confirmation. These included one SNP in the *COL11A1* gene: rs17127311 (difference=-0.0325, p=0.0981). The other five “positive” SNPs were in the *COL18A1* gene: rs2838922 (difference=0.0367, p=0.0572), rs11911327 (difference=-0.0315, p=0.0959), rs2236454 (difference=-0.0510, p=0.0629), rs2236457 (difference=0.0255, p=0.0779), and rs2236475 (difference=-0.0367, p=0.0852). No significant difference in allele frequencies was demonstrated in the remaining 55 SNPs, which were thus not tested any further.

**Table 4:** Pooled DNA analysis of tag SNPs in the *COL11A1*, *COL18A1*, *FBN1*, and *PLOD1* genes.

				**Estimated freq of 1st allele in DNA pools**			
**Candidate gene**	SNP*	**Alleles† (1st/2nd)**	**k correction factor peak height ratio (1st/2nd)**	**Case**	**Control**	**Diff (Case - Control)**	**Nested ANOVA p value**
*COL11A1*	rs2061705	C/T	1.07	0.7361	0.7608	−0.0247	0.3690
	rs2615987	C/A	0.68	0.8228	0.8496	−0.0268	0.1317
	rs17127311 ‡	G/A	0.77	0.1084	0.1409	−0.0325	0.0981
	rs12143740	C/T	1.01	0.1041	0.0929	0.0112	0.6521
	rs4908273	G/A	0.91	0.7256	0.7165	0.0091	0.6941
	rs11164630	G/A	1.02	0.4140	0.3929	0.0211	0.3243
	rs9659030	G/A	1.05	0.2926	0.3079	−0.0153	0.6075
*COL18A1*	rs11089001	C/T	1.07	0.5161	0.5292	−0.0131	0.4837
	rs2026885	G/A	1.09	0.6748	0.6798	−0.0050	0.8748
	rs1004133	G/A	1.56	0.7386	0.7453	−0.0067	0.8369
	rs2838907	C/G	1.14	0.6709	0.6721	−0.0012	0.9681
	rs2838913	G/A	0.29	0.9246	0.9516	−0.0270	0.1642
	rs2838916	G/T	0.97	0.5042	0.5038	0.0004	0.9864
	rs2838917	G/A	0.88	0.5840	0.5627	0.0213	0.4705
	rs2838920	C/T	1.21	0.6460	0.6468	−0.0008	0.5991
	rs2838922 ‡	C/T	1.14	0.7680	0.7313	0.0367	0.0572
	rs2838923	G/A	0.83	0.4811	0.4706	0.0105	0.6356
	rs11911327 ‡	C/T	1.09	0.7656	0.7971	−0.0315	0.0959
	rs8126757	G/A	0.92	0.5004	0.5158	−0.0154	0.4029
	rs11702782	G/A	0.95	0.8379	0.8275	0.0104	0.5183
	rs2026887	C/T	0.94	0.5781	0.5890	−0.0109	0.6956
	rs8129539	C/G	1.17	0.7152	0.6878	0.0274	0.2946
	rs8133622	G/A	1.02	0.3570	0.3572	−0.0002	0.9847
	rs7279077	T/A	1.02	0.2285	0.2334	−0.0049	0.8037
	rs2236454 ‡	C/T	0.57	0.5775	0.6285	−0.0510	0.0629
	rs2236457 ‡	C/T	1.12	0.7031	0.6776	0.0255	0.0779
	rs2230687	C/G	1.17	0.6553	0.6423	0.0130	0.4992
	rs2236475 ‡	G/A	1.14	0.2955	0.3322	−0.0367	0.0852
	rs7279445	C/T	0.86	0.5650	0.5648	0.0002	0.9832
	rs3753019	C/T	1.09	0.6069	0.6164	−0.0095	0.7198
	rs2838951	G/C	1.17	0.4213	0.4260	−0.0047	0.7522
	rs7499	G/A	0.97	0.5045	0.4767	0.0278	0.4194
	rs1051296	G/T	1.14	0.5576	0.5573	0.0003	0.9858
*FBN1*	rs6493334	T/A	0.97	0.6390	0.6384	0.0006	0.9738
	rs6493333	G/A	1.09	0.7846	0.8116	−0.0270	0.1682
	rs6493331	G/T	0.97	0.7304	0.7260	0.0044	0.8532
	rs2247876	G/A	1.06	0.1589	0.1830	−0.0241	0.2860
	rs16961274	C/G	1.09	0.7850	0.7630	0.0220	0.4914
	rs12438332	C/G	1.14	0.8878	0.9112	−0.0234	0.2146
	rs1807301	C/T	1.11	0.3600	0.3805	−0.0205	0.2644
	rs16961207	G/T	1.15	0.7887	0.7972	−0.0085	0.6213
	rs12915677	G/A	1.11	0.6875	0.6756	0.0119	0.6227
	rs683282	G/A	1.06	0.6829	0.6902	−0.0073	0.7380
	rs16961118	G/A	0.89	0.8019	0.7978	0.0041	0.8580
	rs6493328	C/T	1.07	0.5318	0.5100	0.0218	0.3937
	rs17361868	G/A	0.99	0.7825	0.8030	−0.0205	0.1928
	rs11635140	G/A	1.13	0.7071	0.7037	0.0034	0.8172
	rs11070643	G/A	1.00	0.7054	0.6942	0.0112	0.3992
	rs3825963	C/T	1.10	0.7603	0.7988	−0.0385	0.1403
	rs10519174	C/T	0.94	0.6281	0.6329	−0.0048	0.8305
	rs1467953	C/A	1.33	0.6339	0.6361	−0.0022	0.7903
*PLOD1*	rs1208984	G/A	0.97	0.2559	0.2616	−0.0057	0.8053
	rs7529452	C/T	1.03	0.6258	0.5912	0.0346	0.4005
	rs7551175	G/A	1.14	0.7086	0.6950	0.0136	0.6240
	rs11585018	G/A	0.92	0.8047	0.7888	0.0159	0.5827
	rs2273286	G/A	0.99	0.1953	0.1947	0.0006	0.9778
	rs1130529	C/T	0.93	0.5770	0.5978	−0.0208	0.6025
	rs2273291	C/T	1.05	0.6965	0.7189	−0.0224	0.3443
	rs3818157	G/A	1.19	0.3911	0.3726	0.0185	0.4668
	rs3753579	G/A	1.06	0.4766	0.4494	0.0272	0.3591

*SNPs are arranged down the column in the order of 5′>3′ along the sense strand of the respective gene. †The 1st allele has a shorter elution while the 2nd allele has a longer elution time. The alleles are named with reference to the sense strand of the respective gene. ‡SNP that give significant results (p<0.10).

### Confirmation of pooled DNA results by individual genotyping

**Figure 1:**
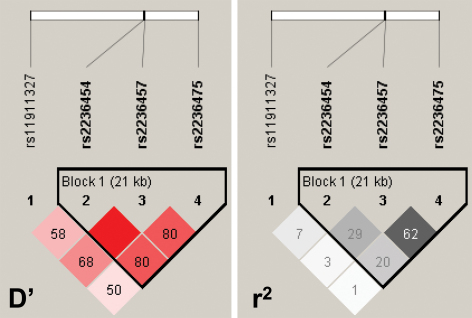
Single nucleotide polymorphisms (SNPs) and their linkage disequilibrium (LD) for the *COL18A1* gene. The SNPs are indicated from the 5′ end (left) to the 3′ end (right) of the gene. The LD measures are expressed as D’ and r^2^ for all subjects under study (cases and controls combined), and are calculated by Haploview. The shades of red (for D’) and gray (for r^2^) represent the magnitude of the measures with deep red equal to 100% (or 1.00), which is omitted in the diagram to avoid cluttering.

The genotypes of these six SNPs were in HWE except two SNPs (rs2838922 and rs223475) in cases and two SNPs (rs17127311 and rs2838922) in controls (Table 5). Deviation from HWE in cases can be a signal for SNP-disease association [25]. The two SNPs violating HWE in controls (rs17127311 and rs2838922) were dropped from subsequent analysis. One haplotype block was constructed for three SNPs as shown in Figure 1. All four SNPs were analyzed for association with high myopia with adjustment for gender, but did not show significant differences in allele frequencies between cases and controls (Table 5). Sliding window-based haplotype analysis of these four SNPs (rs11911327 [S1], rs2236454 [S2], rs2236457 [S3], and rs2236475 [S4] in the 5′>3′ order along the sense strand of the *COL18A1* gene) did not show any association with high myopia either. The p values for the omnibus tests of haplotypes adjusted for gender were as follows: 0.2000 (S1-S2), 0.2850 (S2-S3), 0.1860 (S3-S4), 0.2560 (S1-S2-S3), 0.1840 (S2-S3-S4), and 0.2810 (S1-S2-S3-S4). MDR did not show any significant interaction among the SNPs either, with the best model consisting of all four SNPs (p=0.1719).

**Table 5:** Allelic association tests of *COL11A1* and *COL18A1* SNPs genotyped individually.

** **	Alleles*		Genotype counts (11/12/22)*		**HWE test (p value)**		**Minor allele freq**		**Allelic test†**	
**SNP**	**1**	**2**	**Cases**	**Controls**	**Cases**	**Controls**	**Cases**	**Controls**	**OR (95% CI)**	**p value**
***COL11A1***										
rs17127311	A	G	206/74/5	165/89/2	0.8128	0.0054	0.1474	0.1816	– ‡	– ‡
***COL18A1***										
rs2838922	C	T	197/75/26	174/91/28	4.47E-05	0.0047	0.2131	0.2509	– ‡	– ‡
rs11911327	C	T	197/89/10	192/97/7	1.0000	0.2524	0.1841	0.1875	0.98 (0.72 - 1.33)	0.8979
rs2236454	C	T	74/131/90	79/144/73	0.0621	0.6431	0.5271	0.4899	1.15 (0.92 - 1.44)	0.2132
rs2236457	C	T	183/94/22	180/93/14	0.0508	0.7221	0.2308	0.2108	1.10 (0.84 - 1.44)	0.5011
rs2236475	A	G	184/88/22	176/96/21	0.0187	0.1427	0.2245	0.2355	0.93 (0.71 - 1.20)	0.5672

*The major allele is designated as “1” and minor allele as “2”; and the genotype counts are indicated as the counts of the genotypes 11, 12, and 22, respectively. This study had 300 cases and 300 controls. Note that the total genotype counts may not add up to these expected numbers because a few samples failed to be genotyped in a random fashion. †The allelic test is performed with the gender as a covariate to adjust for the potential confounding by gender because the cases and controls differ significantly in the proportions of male and female subjects (p=4.30×10^–5^).The odds ratio (OR) is calculated for the minor allele (allele 2) with the major allele (allele 1) as the reference. ‡ Association tests are not performed for these two SNPs (rs17127311 and rs2838922) because the genotypes in the controls are not in Hardy–Weinberg equilibrium (HWE).

## Discussion

This study explored the relationship of common polymorphisms in four candidate genes (*COL11A1*, *COL18A1*, *FBN1*, and *PLOD1*) with high myopia in a Han Chinese population. Rare pathogenic mutations in these candidate genes cause disease syndromes that have myopia, usually high myopia, as one of the common presenting features [8-13]. It was logical to investigate whether common polymorphisms of these genes would be predisposing genetic factors for high myopia. This argument was strengthened by the positive association between common polymorphisms of the *COL2A1* gene, the causative gene underlying STL1, and myopia [14,15]. The relationship between the selected candidate genes and high myopia has not been studied before.

With this background, we examined these candidate gene with an efficient approach based on the initial screening of DNA pools. Six case pools and six control pools were constructed and screened by DHPLC analysis of primer extended products. Our case subjects were recruited with a refractive error (SE) threshold of at least −8.00 D for both eyes, and the average SE was −10.53 D. Such a high threshold was adopted for case subject recruitment so as to enrich the contribution of genetic factors to the extreme phenotype and to enhance the homogeneity of the case phenotype [4,6]. Our control subjects were emmetropic and were not randomly recruited from the general Chinese population in Hong Kong. This would also enhance the difference in the genetic components contributing to the phenotype difference between our cases and controls. Random population-based controls were less desirable because they would be enriched with subjects with mild to moderate myopia from our population – a population with a high prevalence of myopia [26]. These strategies would enhance the power of our study.

In the first stage, 60 SNPs from the 4 candidate genes were successfully screened using the DNA pooling approach. Of these, 6 SNPs gave a p value of less than 0.10 for the statistical comparison of allele frequency differences between case pools and control pools by nested ANOVA (Table 4). A lenient significance threshold of p≤0.10 was used to avoid missing potentially significant SNPs. In the second stage, these “positive” SNPs were followed up by individual genotyping, but failed to be confirmed via standard single-marker (Table 5) and haplotype analyses. In conclusion, common polymorphisms in these four candidate genes (*COL11A1*, *COL18A1*, *FBN1*, and *PLOD1*) were unlikely to play important roles in the genetic susceptibility to high myopia.

It is interesting to note that deviations from HWE were observed in the control group for two SNPs (rs17127311 and rs2838922; Table 5). The Hardy–Weinberg principle assumes a very large population in which mating is random and there are no migration, mutation and natural selection [27]. Theoretically, violations of these assumptions can result in deviations from HWE. However, deviations from HWE can indicate the presence of genotyping errors [28]. We had been very careful in carrying out the genotyping and calling the genotypes, and we confirmed any ambiguous genotypes by direct DNA sequencing. However, we cannot entirely rule out the possibility of genotyping errors as a cause for deviations from HWE. It is generally recommended not to perform case-control comparison for such genotype data to avoid false positive association results [28].

We used nested ANOVA [18] to test for differences in estimated allele frequencies between case pools and control pools. Nested ANOVA can properly handle the variance components of the errors arising from sampling of the subjects in forming the pools and of the technical errors arising from various stages of allele frequency estimation, e.g., unequal amounts of individual DNA samples in forming the pools, errors in PCR and primer extension reaction, and in DHPLC analysis. However, the individual variance components could not be estimated directly. Thus, it was not possible to calculate the power of our DNA pooling based-approach, which would expectedly be less than the power of an approach based on genotyping of all individual samples for all tag SNPs. In addition, our DNA pooling strategy did not allow haplotype analysis for SNPs only examined for the DNA pools [16]. On the other hand, we enhanced the power of our study by using stringent criteria for recruiting the cases (extreme phenotype) and the controls (supernormal), as has been discussed above [4,6,26].

DNA pooling has been proven to be effective as an initial screen of SNPs to search for putative genetic markers associated with a phenotype of interest for subsequent follow-up studies based on conventional genotyping of individual samples [16,29]. Saving in the amounts of DNA used and in the cost and time involved in genotyping is the major advantage of DNA pooling. For each SNP, 36 separate PCRs and the following analyses were needed for 6 case pools and 6 control pools together with 3 other separate PCRs for a heterozygous sample to determine the k correction factor. In comparison to genotyping 600 samples individually, our current DNA pooling approach could theoretically reduce the amounts of DNA used and the genotyping work by up to 93.5%. Note that we mixed DNA from 50 distinct individuals to form one DNA pool. This approach of using more pools of smaller size has been shown to be superior to the use of fewer DNA pools each formed from a larger number of subjects for genetic association studies of candidate genes [30].

It may seem that DNA pooling may become less attractive with the recent tremendous reduction in the unit cost of genotyping for high-throughput array-based whole-genome genotyping assays. However, the total cost of such genome-wide genotyping is still too expensive and hence unaffordable for most research groups. Use of DNA pools for genome-wide genotyping is one of the solutions proposed [31]. Recent studies even suggest the use of DNA pools for deep sequencing using next-generation sequencing technologies to explore the role of rare variants in complex diseases [32,33] because both common and rare variants are believed to contribute to the genetic susceptibility to complex diseases [34]. This new development is particularly important because deep sequencing is even more expensive and produces even larger amounts of data for analysis than genome-wide genotyping. Note that the present study did not address the potential role of rare variants that may contribute to the genetic susceptibility to high myopia, but do not cause the respective Mendelian disease syndromes mentioned above. Indeed, there are already rare variants reported to be associated with high myopia but not congenital stationary night blindness ([35] and unpublished data). Congenital stationary night blindness is an X-linked monogenic ocular disease with high myopia as one of its common presenting features [36].

In summary, we examined using a DNA pooling approach tag SNPs from four candidate genes (*COL11A1*, *COL18A1*, *FBN1*, and *PLOD1*) selected because pathogenic mutations in these genes cause disease syndromes that have myopia, usually high myopia, as one of the common presenting clinical features. Six SNPs were followed up by individual genotyping, but did not demonstrate any association with high myopia. We concluded that common polymorphisms in these candidate genes were unlikely to be important in the genetic susceptibility to high myopia in Han Chinese.

## References

[1] Gilmartin B (2004). Myopia: precedents for research in the twenty-first century.. Clin Experiment Ophthalmol.

[2] Saw SM, Gazzard G, Shih-Yen EC, Chua WH (2005). Myopia and associated pathological complications.. Ophthalmic Physiol Opt.

[3] Saw SM, Chua WH, Wu HM, Yap E, Chia KS, Stone RA (2000). Myopia: gene-environment interaction.. Ann Acad Med Singapore.

[4] Tang WC, Yap MK, Yip SP (2008). A review of current approaches to identifying human genes involved in myopia.. Clin Exp Optom.

[5] Young TL, Metlapally R, Shay AE (2007). Complex trait genetics of refractive error.. Arch Ophthalmol.

[6] Hattersley AT, McCarthy MI (2005). What makes a good genetic association study?. Lancet.

[7] Snead MP, Yates JR (1999). Clinical and Molecular genetics of Stickler syndrome.. J Med Genet.

[8] Edwards AO (2008). Clinical features of the congenital vitreoretinopathies.. Eye.

[9] Passos-Bueno MR, Suzuki OT, Armelin-Correa LM, Sertié AL, Errera FI, Bagatini K, Kok F, Leite KR (2006). Mutations in collagen 18A1 and their relevance to the human phenotype.. An Acad Bras Cienc.

[10] Suzuki O, Kague E, Bagatini K, Tu H, Heljasvaara R, Carvalhaes L, Gava E, de Oliveira G, Godoi P, Oliva G, Kitten G, Pihlajaniemi T, Passos-Bueno MR (2009). Novel pathogenic mutations and skin biopsy analysis in Knobloch syndrome.. Mol Vis.

[11] Callewaert B, Malfait F, Loeys B, De Paepe A (2008). Ehlers-Danlos syndromes and Marfan syndrome.. Best Pract Res Clin Rheumatol.

[12] Nahum Y, Spierer A (2008). Ocular features of Marfan syndrome: diagnosis and management.. Isr Med Assoc J.

[13] Yeowell HN, Steinmann B. Ehlers-Danlos Syndrome, Kyphoscoliotic Form. In, Pagon RA, Bird TD, Dolan CR, Stephens K, editors. GeneReviews. Seattle (WA): University of Washington, Seattle; 1993–2000. 20301635

[14] Mutti DO, Cooper ME, O'Brien S, Jones LA, Marazita ML, Murray JC, Zadnik K (2007). Candidate gene and locus analysis of myopia.. Mol Vis.

[15] Metlapally R, Li YJ, Tran-Viet KN, Abbott D, Czaja GR, Malecaze F, Calvas P, Mackey D, Rosenberg T, Paget S, Zayats T, Owen MJ, Guggenheim JA, Young TL (2009). COL1A1 and COL2A1 genes and myopia susceptibility: evidence of association and suggestive linkage to the COL2A1 locus.. Invest Ophthalmol Vis Sci.

[16] Sham P, Bader JS, Craig I, O'Donovan M, Owen M (2002). DNA Pooling: a tool for large-scale association studies.. Nat Rev Genet.

[17] Hoogendoorn B, Norton N, Kirov G, Williams N, Hamshere ML, Spurlock G, Austin J, Stephens MK, Buckland PR, Owen MJ, O'Donovan MC (2000). Cheap, accurate and rapid allele frequency estimation of single nucleotide polymorphisms by primer extension and DHPLC in DNA pools.. Hum Genet.

[18] Zar JH. Biostatistical Analysis, 5th ed. New Jersey: Prentice Hall/Pearson; 2010. p. 307–27.

[19] Zha Y, Leung KH, Lo KK, Fung WY, Ng PW, Shi MG, Yap MK, Yip SP (2009). TGFB1 as a susceptibility gene for high myopia: a replication study with new findings.. Arch Ophthalmol.

[20] de Bakker PI, Yelensky R, Pe'er I, Gabriel SB, Daly MJ, Altshuler D (2005). Efficiency and power in genetic association studies.. Nat Genet.

[21] Han W, Yip SP, Wang J, Yap MK (2004). Using denaturing HPLC for SNP discovery and genotyping, and establishing the linkage disequilibrium pattern for the all-trans-retinol dehydrogenase (RDH8) gene.. J Hum Genet.

[22] Purcell S, Neale B, Todd-Brown K, Thomas L, Ferreira MA, Bender D, Maller J, Sklar P, de Bakker PI, Daly MJ, Sham PC (2007). PLINK: a tool set for whole-genome association and population-based linkage analyses.. Am J Hum Genet.

[23] Barrett JC, Fry B, Maller J, Daly MJ (2005). Haploview: analysis and visualization of LD and haplotype maps.. Bioinformatics.

[24] Moore JH, Gilbert JC, Tsai CT, Chiang FT, Holden T, Barney N, White BC (2006). A flexible computational framework for detecting, characterizing, and interpreting statistical patterns of epistasis in genetic studies of human disease susceptibility.. J Theor Biol.

[25] Nielsen DM, Ehm MG, Weir BS (1998). Detecting marker-disease association by testing for Hardy-Weinberg disequilibrium at a marker locus.. Am J Hum Genet.

[26] Edwards MH, Lam CS (2004). The epidemiology of myopia in Hong Kong.. Ann Acad Med Singapore.

[27] Hedrick PW. Genetics of Populations, 4th ed. Sudbury: Jones and Barlett; 2011.

[28] Hosking L, Lumsden S, Lewis K, Yeo A, McCarthy L, Bansal A, Riley J, Purvis I, Xu CF (2004). Detection of genotyping errors by Hardy-Weinberg equilibrium testing.. Eur J Hum Genet.

[29] Bansal A, van den Boom D, Kammerer S, Honisch C, Adam G, Cantor CR, Kleyn P, Braun A (2002). Association testing by DNA pooling: an effective initial screen.. Proc Natl Acad Sci USA.

[30] Barratt BJ, Payne F, Rance HE, Nutland S, Todd JA, Clayton DG (2002). Identification of the sources of error in allele frequency estimations from pooled DNA indicates an optimal experimental design.. Ann Hum Genet.

[31] Macgregor S, Zhao ZZ, Henders A, Nicholas MG, Montgomery GW, Visscher PM (2008). Highly cost-efficient genome-wide association studies using DNA pools and dense SNP arrays.. Nucleic Acids Res.

[32] Price AL, Kryukov GV, de Bakker PI, Purcell SM, Staples J, Wei LJ, Sunyaev SR (2010). Pooled association tests for rare variants in exon-resequencing studies.. Am J Hum Genet.

[33] Wang T, Lin CY, Rohan TE, Ye K (2010). Resequencing of pooled DNA for detecting disease associations with rare variants.. Genet Epidemiol.

[34] Bodmer W, Bonilla C (2008). Common and rare variants in multifactorial susceptibility to common diseases.. Nat Genet.

[35] Zhang Q, Xiao X, Li S, Jia X, Yang Z, Huang S, Caruso RC, Guan T, Sergeev Y, Guo X, Hejtmancik JF (2007). Mutations in NYX of individuals with high myopia, but without night blindness.. Mol Vis.

[36] Miyake Y, Yagasaki K, Horiguchi M, Kawase Y, Kanda T (1986). Congenital stationary night blindness with negative electroretinogram. A new classification.. Arch Ophthalmol.

